# Towards inclusive health research: the added value of making invitation letters and questionnaires available in minority languages

**DOI:** 10.1016/j.jmh.2026.100454

**Published:** 2026-10-27

**Authors:** Lili L. Kókai, Carel-Peter L. van Erpecum, Famke J.M. Mölenberg, Anna Bornioli, Süleyman Adahi Şahin, Pilar Garcia-Gomez

**Affiliations:** a Department of Public Health, Erasmus MC, Rotterdam, The Netherlands; b Division of Human Nutrition and Health, Wageningen University & Research, Wageningen, The Netherlands; c Erasmus School of Economics, Tinbergen Institute and Erasmus Centre for Health Economics Rotterdam, Rotterdam, The Netherlands; d ISGLOBAL Institute of Global Health Barcelona, Barcelona, Spain; e Civil Engineering Department, Sakarya University of Applied Sciences, Sakarya, Turkey

**Keywords:** Minority languages, Migration background diversity, Socioeconomic diversity, Inclusive recruitment strategy, Health equity

## Abstract

**Objectives:**

To pilot whether providing study materials in minority languages is associated with an improved inclusion of underrepresented migrant and socioeconomic populations in health research, and an increased willingness of these populations to be contacted for future research.

**Methods:**

We analyzed two waves of a repeated cross-sectional questionnaire in Rotterdam, the Netherlands. In wave 1, materials were available in Dutch and English. In wave 2, Arabic and Turkish - the most prevalent minority languages - were added. We compared the share of participants with a migration background from an Arabic-speaking country or Turkey between waves and described socioeconomic characteristics by survey language (total n = 1164; n = 557 in wave 1 and n = 607 in wave 2). Among participants willing to be contacted for future research, migration background was compared between waves (total n = 440; n = 206 in wave 1 and n = 234 in wave 2).

**Results:**

The share of participants with a first-generation migration background from Turkey increased from 1.4% in wave 1 to 4.1% in wave 2. Socioeconomic diversity increased: 46.7% respondents choosing Standard Arabic and 33.1% of respondents choosing Turkish had lower- or middle-level education. Among participants willing to be contacted for future research, the share with a first-generation migration background from Turkey rose from 1.9% to 6.4%.

**Conclusions:**

Including minority language materials in health surveys is feasible and is associated with improved representativeness, supporting equitable health research and policymaking. Future efforts need to integrate additional culturally sensitive strategies.

## 1 Introduction

Minority groups are underrepresented in research and one reason for this is a language barrier (Pardhan et al., 2025). This is an especially important issue in health-related research, as people with a migration background may have worse health, even when controlling for education and income (Agyemang, 2019). Some studies suggest that people with a migration background may have lower socioeconomic status; then, inclusive recruitment and data collection strategies would also increase socioeconomic diversity in participants (Füller et al., 2023).

With minority populations increasing (de Regt, 2021), it is crucial that we manage to recruit and sustain individuals from these backgrounds in scientific studies. If we fail to do so, policy makers and other health-related professionals will not have an accurate view of the health of a large part of the population, and consequently will not be able to meet their needs and preferences and support their trust in public institutions, thereby contributing to population-level health disparities. Indeed, willingness to take part in future research is an established indicator of trust in science (Hu et al., 2023).

The aim of the current study was to assess if making research invitations and questionnaires available in the two most prevalent minority languages in the Netherlands - Arabic and Turkish - in addition to Dutch and English materials is associated with an increase in 1) the inclusion of underrepresented migrant and socioeconomic populations in health research and 2) the willingness of these populations to be contacted for future research.

## 2 Methods

### 2.1 Context

Rotterdam is an industrial city in the Netherlands with approximately half of its 650,000 inhabitants having a migration background. A migration background from Morocco or Turkey is the most common - approximately 7% each of the whole city’s population - with much of the first generation having migrated in the 1960s (Penninx et al., 1993; Statistiek, 2023).

A study found that the share of individuals experiencing difficulty with the Dutch language when reading, writing and/or speaking was 39% for people with a first-, and 5% for people with a second-generation migration background from Morocco; and 51% for people with a first-, and 4% for people with a second-generation migration background from Turkey (Hogendoorn, 2023).

### 2.2 Study design

#### 2.2.1 Host study

The current pilot study is based in a study in Rotterdam designed to investigate the broader health impacts of 30km/h speed limit interventions. A study protocol has been published elsewhere (van Erpecum et al., 2025). Some addresses were selected in a street where the speed limit changed from 50 km/h to 30 km/h, and others - where the speed limit did not change but were similar streets in terms of address density and neighborhood socioeconomic status - were identified as controls. Both intervention and control group received a questionnaire invitation prior to (wave 1) and after (wave 2) the speed limit change in the intervention group.

#### 2.2.2 Wave 1

An invitation to a 15-minute repeated cross-sectional household questionnaire was distributed to 10,860 addresses in central areas of Rotterdam between June and August of 2023 in Dutch and English. The invitation described the research (healthy neighborhoods) and mentioned that three vouchers of 100 euros would be randomly gifted to some participants. Participants used a QR-code in the invitation to fill out an online consent form and questionnaire in Dutch or English. Participants could also request a paper-and-pencil version of the questionnaire. The questionnaire asked about sociodemographic characteristics, health and social outcomes, lifestyle, and willingness to be contacted to participate in future research.

#### 2.2.3 Wave 2

The study used a repeated cross-sectional design. Consequently, at wave 2 between September and November 2024, the same questionnaire was sent to the same 10,860 addresses as at wave 1, rather than only to individuals that already participated in the study at baseline. Multiple individuals from the same household could participate in the survey, and a small proportion did so in wave 1 (n = 70; 12.6%) and in wave 2 (n = 76; 12.5%). In wave 2, we added a Standard Arabic and a Turkish version of the invitation letter and questionnaire. Translations to Standard Arabic were performed by a professional translation agency (Acolad Utrecht). Translations to Turkish was performed by a member of the research team (SAS) whose mother tongue is Turkish and had previous work experience in translating English research materials to Turkish. Invitation delivery, questionnaire administration, eligibility criteria, reminders, incentives, duration of data collection and procedures for accessing and completing the questionnaire were identical for the two waves.

### 2.3 Outcomes

#### 2.3.1 Migration background

Migration background was defined based on the country of birth of the participant and that of their mother and father (Statistiek, 2021a). Participants could fill out one of the following options for their country of birth and that of their parents: the Netherlands, Morocco, Turkey, Suriname, Dutch Caribbean, Indonesia, “Other, namely…”, or “Prefer not to say”. We categorized participants into the following migration backgrounds: the Netherlands, non-Netherlands Western (European countries excluding Turkey; North American and Oceanian countries, Indonesia and Japan (Statistiek, 2021b)), Morocco or other country with Arabic as official language (i.e., Algeria, Bahrain, Chad, Djibouti, Egypt, Eritrea, Iraq, Jordan, Kuwait, Lebanon, Mauritania, Oman, Palestine, Saudi Arabia, Sudan, Syria, Tunisia, United Arab Emirates, or Yemen), or other non-Western countries. We merged Morocco and other countries with Arabic as official language because of the small numbers in both groups. We distinguished between people with a first-generation (individual born in another country than the Netherlands) or a second-generation (parent(s) of individual born in another country than the Netherlands) migration background.

#### 2.3.2 Willingness to be contacted to participate in future research

Willingness to be contacted to participate in future research was assessed with: “We are thinking of conducting short interviews and/or invite residents in Rotterdam to wear a physical activity tracker to measure physical activity. This will help us even more to create healthier neighborhoods in Rotterdam! Would it be possible to recontact you for future research by our research team?”

#### 2.3.3 Sociodemographic characteristics

Age (in years) and gender (female, male, or other) were assessed.

Socioeconomic status was measured by educational level (lower, middle, or higher, as per the International Standard Classification of Education 2011 (Eurostat, 2011)) and employment status (working full-time (i.e., 5 days a week), working part-time (i.e., 1–4 days per week), no paid job, study/education, other).

### 2.4 Study sample

We used the data of participants who had complete responses on migration background, age, gender, educational level and employment status (hereon referred to as ‘questionnaire participants’; total n = 1164; n = 557 in wave 1 and n = 607 in wave 2), and a subset of these participants who additionally indicated to be willing to be contacted to participate in future research (hereon referred to as ‘questionnaire participants willing to be contacted to participate in future research’; total n = 440; n = 206 in wave 1 and n = 234 in wave 2).

### 2.5 Statistical analysis

Given the small sample sizes, we did not perform statistical testing. We descriptively compared the share of participants with a migration background from an Arabic-speaking country or Turkey between waves, and described age, gender, educational level and employment status by chosen survey language. Among participants willing to be contacted for future research, migration background was compared between waves.

## 3 Results

The share of *questionnaire* participants with a migration background from an Arabic-speaking country or Turkey before and after the use of the inclusive strategy is shown in Fig. 1a. The share of participants with a first-generation migration background from Turkey increased from 1.4% (n = 8) to 4.1% (n = 25). There was no meaningful change in the share of participants with a second-generation migration background from Turkey (1.8% (n = 10) to 1.5% (n = 9)), or from an Arabic-speaking country (first-generation: 1.4% (n = 8) to 0.8% (n = 5); second-generation: 0.2% (n = 1) to 0.0% (n = 0)).

**Fig. 1:**
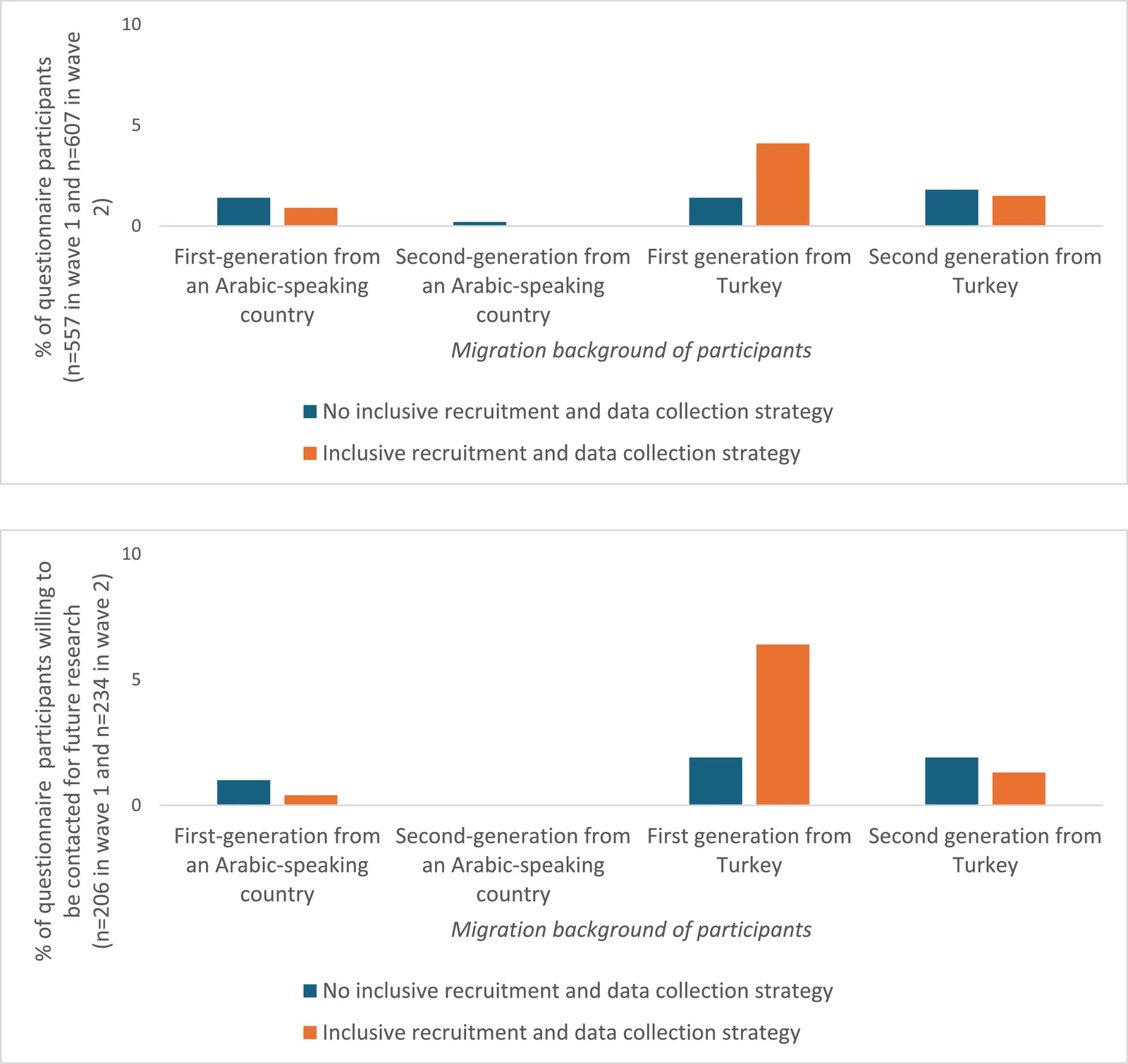
1a and 1b. Share of participants with a migration background from an Arabic-speaking country or Turkey by recruitment and data collection strategy. First and second-generation migration backgrounds are shown separately. Data of questionnaire participants and questionnaire participants (above, Fig. 1a) and willing to be contacted for future research (below, Fig. 1b) are also shown separately.

The share of questionnaire participants *willing to be contacted to participate in future research* with a migration background from an Arabic-speaking country or Turkey before and after the addition of the inclusive strategy is shown in Fig. 1b. Patterns were similar to those in the questionnaire participants, with the share of participants with a first-generation migration background from Turkey increasing, from 1.9% (n = 4) to 6.4% (n = 15). The share of participants with a second-generation migration background from Turkey, or a first- or second-generation migration background from an Arabic-speaking country did not change meaningfully (1.9% (n = 4) to 1.3% (n = 3); 1.0% (n = 2) to 0.4% (n = 1); and 0.0% (n = 0) to 0.0% (n = 0), respectively).

In Table 1, the sociodemographic characteristics of participants filling out the questionnaire in different languages is shown. In the second wave, the percentage of participants that filled out the questionnaire in Turkish and Arabic were 2.5% (n = 15) and 0.5% (n = 3), respectively. Importantly, the increase in the share of participants with a first-generation migration background from Turkey who filled out the second questionnaire in Turkish was not accompanied by a decrease in the share of participants with this migration background who chose to fill in the questionnaire in Dutch (0.8% (n = 4) in the first wave to 1.0% (n = 5) in the second wave) or English (4.7% (n = 4) to 5.5% (n = 6), respectively). The share of participants in the second wave with a lower- or a middle-level education among those that filled in the questionnaire in Turkish, Standard Arabic, Dutch or English was 46.7% (n = 7), 33.1% (n = 1), 21.2% (n = 104), and 8.1% (n = 9), respectively. Of all participants in the second wave with a lower educational level, 23.1% (n = 3) filled out the questionnaire in Turkish.

**Table 1:** Sociodemographic characteristics of participants filling out the questionnaire in different languages.

	Questionnaire language at the first wave				Questionnaire language at the second wave			
	*Dutch*	*English*	*Turkish*	*Standard Arabic*	*Dutch*	*English*	*Turkish*	*Standard Arabic*
*n (%)*^1^	472 (84.7)	85 (15.3)	NA	NA	479 (78.9)	110 (18.1)	15 (2.5)	3 (0.5)
*Age, mean (SD)*	50.9 (17.4)	36.8 (11.3)	NA	NA	49.6 (17.5)	35.7 (11.1)	46.6 (10.5)	50.0 (21.4)
*Gender, female, n (%)*	273 (57.8)	42 (49.4)	NA	NA	260 (54.3)	62 (56.4)	5 (33.3)	2 (66.7)
*Migration background*								
*The Netherlands, n (%)*	341 (72.2)	3 (3.5)	NA	NA	362 (75.6)	10 (9.1)	0 (0.0)	0 (0.0)
*First-generation from Arabic-speaking country, n (%)*	5 (1.1)	3 (3.5)	NA	NA	3 (0.6)	0 (0.0)	0 (0.0)	2 (66.7)
*Second-generation from Arabic-speaking country, n (%)*	1 (0.2)	0 (0.0)	NA	NA	0 (0.0)	0 (0.0)	0 (0.0)	0 (0.0)
*First-generation from Turkey, n (%)*	4 (0.8)	4 (4.7)	NA	NA	5 (1.0)	6 (5.5)	14 (93.3)	0 (0.0)
*Second-generation from Turkey, n (%)*	10 (2.1)	0 (0.0)	NA	NA	8 (1.7)	0 (0.0)	1 (6.7)	0 (0.0)
*First-generation from non-Netherlands Western country, n (%)*	20 (4.1)	37 (43.5)	NA	NA	21 (4.4)	56 (50.9)	0 (0.0)	0 (0.0)
*Second-generation from non-Netherlands Western country, n (%)*	14 (3.0)	2 (2.4)	NA	NA	19 (4.0)	1 (0.9)	0 (0.0)	0 (0.0)
*First-generation from other non-Western country, n (%)*	32 (6.8)	35 (41.2)	NA	NA	24 (5.0)	34 (30.9)	0 (0.0)	1 (33.3)
*Second-generation from other non-Western country, n (%)*	22 (4.7)	0 (0.0)	NA	NA	30 (6.3)	3 (2.7)	0 (0.0)	0 (0.0)
*Other or prefer not to say, n (%)*	23 (4.9)	1 (1.2)	NA	NA	7 (1.5)	0 (0.0)	0 (0.0)	0 (0.0)
*Educational level*								
*Lower, n %)*	11 (2.3)	0 (0.0)	NA	NA	9 (1.9)	1 (0.9)	3 (20.0)	0 (0.0)
*Middle, n (%)*	95 (20.1)	6 (7.1)	NA	NA	95 (19.8)	8 (7.3)	4 (26.7)	1 (33.3)
*Higher, n (%)*	349 (73.9)	72 (84.7)	NA	NA	362 (75.6)	96 (87.3)	6 (40.0)	1 (33.3)
*Other, n (%)*	17 (3.6)	7 (8.2)	NA	NA	13 (2.7)	5 (4.5)	2 (13.3)	1 (33.3)
*Work situation*								
*Full-time job, n (%)*	228 (48.3)	55 (64.7)	NA	NA	207 (43.2)	80 (72.7)	11 (73.3)	3 (100)
*Part-time job, n (%)*	121 (25.6)	10 (11.8)	NA	NA	137 (28.6)	14 (12.7)	0 (0.0)	0 (0.0)
*No paid job, n (%)*	44 (9.3)	7 (8.2)	NA	NA	39 (8.1)	4 (3.6)	3 (20.0)	0 (0.0)
*In study/education, n (%)*	21 (4.4)	15 (17.6)	NA	NA	28 (5.8)	12 (10.9)	2 (13.3)	0 (0.0)
*Other, n (%)*	102 (21.6)	4 (4.7)	NA	NA	107 (22.3)	4 (3.6)	2 (13.3)	0(0.0)

1 : Percentages in this row are based on the n in the relevant wave. All other percentages in the table are based on the n in the relevant wave and questionnaire language (e.g., wave 1, Dutch).

## 4 Discussion

The current study assessed the association between an inclusive recruitment and data collection strategy and the inclusion of underrepresented migrant and socioeconomic populations in health research, and the willingness of these populations to be contacted for future research. The results of this study suggest that offering research invitations and questionnaires in minority languages may be associated with an improved inclusion of underrepresented migrant and socioeconomic populations, and an increased willingness of these populations to be contacted for future research. In our study this was the case by reaching individuals with a first-generation migration background from Turkey. It makes sense that our strategy would impact first-generation migrants more, given that the host language tends to be more challenging for them than for second-generation migrants (Hogendoorn, 2023). Participants that completed the questionnaires in Turkish or Arabic generally had a relatively lower education, suggesting that such inclusive strategies could also diversify the socioeconomic distribution of research samples. Furthermore, among those willing to be contacted to participate in future research, the proportion of participants with a first-generation migration background from Turkey rose notably from the first to the second wave. These are encouraging indications that modest adaptations in recruitment and study materials can lower barriers for participation for some populations underrepresented in health research.

Our findings, however, show no increase in participation among individuals with a second-generation migration background from Turkey, or with a first- or second-generation migration background from an Arabic-speaking country. Possible explanations include that Standard Arabic is not the same as the Arabic that people use day-to-day; and lower trust and/or interest in research. These differences underline that linguistic accessibility alone may not be sufficient to recruit and retain all communities - future efforts need to integrate additional culturally sensitive strategies, including community outreach through trusted cultural and religious institutions, oral questionnaire completion options, and other personalized approaches (Pardhan et al., 2025). A best practice when it comes to inclusivity in social surveys is the Ethnic Minority Boost Sample within the population-representative study Understanding Society in the UK (Lynn et al., 2018). The boosting aims at reaching statistical power for major ethnic minority groups, achieved through geographic screening. For studies in which such boosted recruitment is not viable, the strategy applied in the current study may be a pragmatic solution to lower barriers for participation for underrepresented migrant and socioeconomic populations.

Several limitations must be acknowledged. First, the translation process for the Turkish and Standard Arabic materials did not follow a formal forward–backward protocol due to time and resource constraints; arguably strengthening the external validity and scalability of our methods. Second, the modest number of participants choosing to fill in the questionnaire in a minority language— especially Standard Arabic — precludes hypothesis testing; further, it limits statistical generalizability, as well as the certainty that we can have regarding our conclusions on the association between translated questionnaires and an increased (willingness for) participation of underrepresented populations. Fourth, as 15 months have passed between the two waves, temporal changes in the composition of the local migrant population or other contextual factors may have contributed to the observed differences. Lastly, the increase in participants with a migration background from Turkey should be interpreted in the context of Rotterdam’s unique demographic composition and cannot be assumed to generalize without adaptation.

Despite these limitations, this study underscores the feasibility and added value of integrating minority language materials in health survey design. In a time when trust in institutions varies widely across population groups, inclusive and accessible research tools can help capture more representative voices and support more equitable health research and policymaking.

## Declarations

### Consent to participate

Informed consent was obtained from all individual participants included in the study.

### Funding

Vital Cities and Citizens (Erasmus University Rotterdam) – no grant number available.

### Ethics approval

The study was conducted in accordance with the principles of the Declaration of Helsinki (Association, 2013). The protocol of the study was approved by the local ethics committee of the Erasmus School of Economics, Rotterdam, the Netherlands (protocol number ETH2223–0833).

### Data sharing statement

Anonymized data will be made available at the Erasmus University open research data repository.

### Clinical trial

Not applicable.

## Ethics declaration

Written informed consent to take part in the study and to publish the article has been obtained from all participants or their legal representatives. The privacy rights of participants have been observed.

This study was performed in compliance with relevant laws, regulatory frameworks and guidelines where the research took place. This study was conducted in accordance with The study was conducted in accordance with the principles of the Declaration of Helsinki. This study was approved by the Erasmus School of Economics, Rotterdam, the Netherlands. (Approval No. Protocol number ETH2223–0833)

## CRediT authorship contribution statement

**Lili L. Kókai:** Writing – review & editing, Writing – original draft, Methodology, Funding acquisition, Conceptualization. **Carel-Peter L. van Erpecum:** Writing – review & editing, Writing – original draft, Methodology, Funding acquisition, Formal analysis, Conceptualization. **Famke J.M. Mölenberg:** Writing – review & editing, Writing – original draft, Methodology, Funding acquisition, Conceptualization. **Anna Bornioli:** Writing – review & editing, Funding acquisition, Conceptualization. **Süleyman Adahi Şahin:** Writing – review & editing, Methodology. **Pilar Garcia-Gomez:** Writing – review & editing, Funding acquisition, Conceptualization.

## Declaration of competing interest

The authors declare to have no competing interests.
